# Pai te moe, pai te ora: exploring the sociocultural practice of sleep in Aotearoa New Zealand through Māori media

**DOI:** 10.1093/heapro/daag034

**Published:** 2026-03-17

**Authors:** Deanna Haami, Zoe Pullman, Rosemary Gibson, Natasha Tassell-Matamua

**Affiliations:** School of People, Environment and Planning, Massey University (PN331), Private Bag 11 222, Palmerston North 4442, Manawatū-Whanganui, New Zealand; School of Psychology, Massey University, Private Bag 11-222, Palmerston North 4442, Manawatū-Whanganui, New Zealand; School of Psychology, Massey University, Private Bag 11-222, Palmerston North 4442, Manawatū-Whanganui, New Zealand; Centre for Indigenous Psychologies, School of Psychology, Massey University, Private Bag 11-222, Palmerston North 4442, Manawatū-Whanganui, New Zealand

**Keywords:** sleep, sleep health, media analysis, Indigenous wellbeing, social discourses

## Abstract

Media has the potential to influence beliefs and social practices regarding sleep health. Sponsored articles, amateur guidance, and product advertising sit alongside Western scientific sleep research, which privileges biological and medicalized conceptualizations. Within this space, Indigenous knowledge and perspectives have largely been absent, creating a gap in culturally grounded understandings of sleep as a social practice. To begin the process of reclaiming and re-privileging Indigenous Māori sleep knowledge, 270 media texts concerning sleep were sourced from online media content predominantly created by and for Indigenous Māori in Aotearoa New Zealand (AoNZ). Texts were analysed thematically and organized in accordance with the Māori model of wellbeing Te Whare Tapa Whā (referring to the four-sided house, a holistic analogy of wellbeing). Five themes are presented: Te Taha Wairua, sleep as a spiritual experience; Te Taha Hinengaro, relationship between sleep and psychological wellbeing; Te Taha Tinana, relationship between sleep and physical wellbeing; Te Taha Whānau, sleep as a shared experience; and Te Taha Whenua, a place and space to sleep. Together, these illustrate key content concerning the understanding of sleep as a social and cultural practice for Māori in AoNZ. This research informs the reclamation and re-privileging of Indigenous sleep knowledge, aiding the development of future research and health promotion practices that are culturally responsive.

Contribution to Health Promotion

- Media dissemination of sleep health research shapes ideas of what constitutes a ‘good sleep’, influencing behaviours and practices to attain this ideal.
- The majority of sleep health research prioritizes Western, biomedical perspectives, resulting in media messaging that excludes Indigenous knowledge, perspectives, and practices.
- Indigenous knowledge, perspectives, and practices regarding what makes a sleep ‘good’ often contrasts with what is idealized in sleep health research as the ‘right’ behaviours and practices for attaining a ‘good sleep’.
- Indigenous-led research and media is vital for the decolonization of sleep health, ensuring more relevant, reflective, and holistic messaging.

## Introduction

Indigenous perspectives of health are increasingly called on for holistic appreciations of wellness (Wilson *et al.* 2021). Within such perspectives, sleep is viewed as necessary for attaining and maintaining holistic wellbeing, vital to the spiritual, psychological, physical, and familial dimensions of health (Lindsay *et al.* 2022, Muller *et al.* 2023, Haami *et al.* 2024b). Although the historical and ongoing colonizing agenda in Aotearoa New Zealand (AoNZ) has influenced the way ‘Mātauranga Māori’—the extensive repository of knowledge developed by Māori over thousands of years (Hikuroa 2017)—is transmitted, for many Māori (the Indigenous people of AoNZ), sleep continues to be positioned as a ‘wairua’ (referring to the spirit and the spiritual dimension of lived reality) experience or state that influences the wellbeing of all other dimensions of health (Lindsay *et al.* 2022, Haami *et al.* 2024b). This is perhaps best exemplified in the ‘tikanga’ (culturally informed protocols or practices developed to keep the collective safe) of not waking a sleeping person due to the belief that their wairua travels away from their body during sleep, journeying across time and space. Thus, waking them is considered dangerous as their wairua may not have time to return (Best 1922). Furthermore, activities around the patterns of sleep continue to be informed by tikanga, with preferences for co-sleeping and structuring bedtimes around family rather than work (Lindsay *et al.* 2022, Crestani *et al.* 2024). Furthermore, periods of wakefulness in the night are often reported as positive opportunities for engaging with wairua or creative thought rather than disorder (Crestani *et al.* 2024, Haami *et al.* 2024a).

Such perspectives are important to consider in an era where Western scientific perspectives have colonized sleep, privileging a biomedical lens and leaving little room for alternative interpretations (Williams 2002, 2011). The science of sleep has been grounded Western Academic Scientific Psychology (WASP) (Berry 2015), which privileges biomedical perspectives and approaches to understanding sleep and addressing sleep health (Dement 2008). Research from Western, Educated, Industrialised, Rich, and Democratic (WEIRD) peoples and societies—specifically settler colonial societies where those of white, Euro-Christian descent are privileged—dominate the discourse, which typically defines, evaluates, and treats sleep problems (Henrich *et al.* 2010). Sleep science in AoNZ has followed a similar course, taking a predominantly epidemiological and clinical focus (Ross *et al.* 2024). Such approaches have highlighted societal inequities of sleep, with Māori populations identified as being significantly more likely to have disordered sleep (e.g. Paine *et al.* 2005, Mihaere *et al.* 2009, Gibson *et al.* 2020); higher rates of sudden unexpected death in infancy (SUDI) (Houkamau and Clarke 2016); and more likely to experience challenging sleep environments, such as homelessness and overcrowded housing (Boulton *et al.* 2022). Such disparities in sleep health have been associated with inequities in socioeconomics, compromised sleeping conditions, and limited or inappropriate healthcare (Paine *et al.* 2004, Paine and Gander 2013, 2016, Muller *et al.* 2020, McLay *et al.* 2023)—all of which originates within colonization (Reid *et al.* 2014). However, culture, living environments, family, and wider society also play a role in sleep-related expectations and practices (Williams 2007, Staton *et al.* 2019, Billings *et al.* 2020). A wider sociological lens and the reclamation and re-privileging of Indigenous sleep knowledge are required to fully understand sleep as a social and cultural practice for Māori in AoNZ.

A scoping review by Ross *et al.* (2024) exploring sleep as a sociocultural practice in AoNZ highlighted the need for Indigenous knowledge, perspectives, and practices to decolonize and modernize frameworks of sleep. Since this review, there have been several notable contributions addressing this gap. For example, Haami *et al.* (2024) used a ‘whakapapa’ (genealogical origins or layers of history) thematic analysis of interviews to construct a spiritual explanatory framework for the sleep experiences of Māori. Sleep was described as providing the opportunity for ‘tohu’ (guidance), ‘ako’ (space and time for learning), and ‘tau’ (attaining a state of stability, peace, and purpose). Similarly, group interviews with older Māori identified the appreciation for the spiritual as well as somatic role of sleep states and practices; the changing obligations around sleep and wake across the lifespan; and culturally informed approaches to supporting sleep (Gibson *et al.* 2024). Such works demonstrate the multifaceted nature of sleep and culturally relevant interpretations beyond typical WEIRD models. However, this work should be considered as foundational steps toward the reclamation of ‘Mātauranga Moe’ (repository of knowledge related to sleep), as the diversity of sleep norms, perspectives, and practices for Māori have yet to be fully represented.

Media has the potential to reflect as well as effect beliefs and practices around time use and health behaviours, including sleep (Dew *et al.* 2016). Sponsored articles, amateur guidance, and product advertising sit alongside research findings and real-life accounts. Media content can, therefore, entrench broader discourses and stigmas relating to sleeplessness and disease as well as age, gender, class, and ethnicity. Previous media-related sleep research has mostly been Eurocentric, focused on the medicalization of sleep problems and treatments (e.g. Boden *et al.* 2008, Varallo *et al.* 2022), gendered constructs of sleep (Zarhin 2021), and fitting sleep in around working life (Boden *et al.* 2008 ). These studies indicate that media messages are often divorced from both academic research and the realities of individual and social circumstances. Recent AoNZ studies have considered the portrayal of sleep during pregnancy and sleep among older people within mainstream news (Ladyman *et al.* 2022, Breheny *et al.* 2023). It was found that, while some of these media texts were informative and useful, many were less accurate, commercialized, or sensationalized. This is of concern given the power the media has to shape narratives to align with specific worldviews—such as that derived from Western, colonial paradigms—that then influence societal beliefs and practices. This is particularly relevant for health messaging, which is often dominated by a singular worldview, resulting in further harm and continuing to perpetuate health inequities. This is exemplified in the messaging around co-sleeping and bed sharing as being ‘unhealthy’ and a ‘poor sleep habit’. Co-sleeping has been a central sleep practice for many Indigenous cultures across the world since before colonization yet is now positioned as a reflection of ‘bad parenting’. This messaging then prevents parents engaging with health services and professionals fully for fear of judgement, as it is this Western paradigm that acts as the lens for understanding and interpreting their parenting (George *et al.* 2020, 2021). Furthermore, only around 20% of articles published in AoNZ’s mainstream media refer to Māori, though these are not necessarily authored by Māori, for Māori, or relevant to Mātauranga Māori (Nairn *et al.* 2006). The need for broader approaches has been identified, using alternative media sources centred in ‘Te Ao Māori’ (Māori worldview), to collate and represent the true diversity of sleep as a practice in AoNZ (Gibson 2025).

Given the gaps highlighted above, the aim of the present research was to more fully understand sleep as a social and cultural practice in contemporary AoNZ from an Indigenous Māori perspective. Online media content predominantly created by and for Māori was explored regarding the topic of sleep to provide a culturally informed overview of the discourses of sleep within AoNZ media. This will extend pre-existing Western models of sleep and aid development of future sleep resources that are responsive to Māori.

## Materials and methods

### Methodology

This research was guided by the principles of ‘Kaupapa Māori’ research, which, at its essence, privileges research that is by Māori, for Māori, and with Māori (Smith 2015) while also recognizing the diverse realities of Māori in contemporary times (Durie 1995, Moewaka Barnes 2008). Throughout the research, the Māori members of the research team led the search, collection, analysis, and interpretation of the data, ensuring the media content was consistently considered through a Te Ao Māori lens. A key part of this was ensuring the inclusion of te reo Māori (referring to the Māori language) words and conceptualizations of sleep, ensuring the nuances of the sleep experience for Māori was captured.

### Ethical approval

Data were derived from pre-existing sources in the public domain rather than from human participants. The research was conducted according to the ethical principles of the Massey University Human Ethics Committee.

### Search strategy and data collection

**Figure 1:**
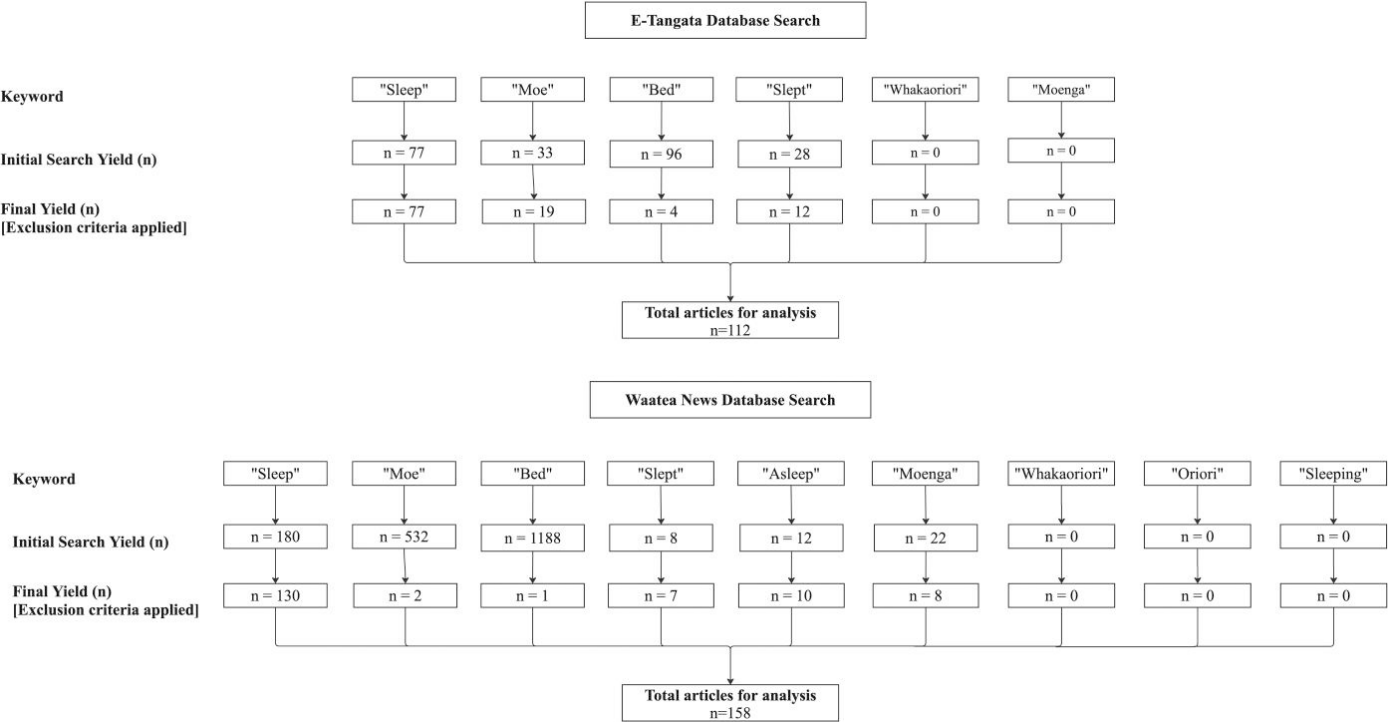
Search results from E-Tangata and Waatea News

Two Māori media outlets, ‘E-Tangata’, a not-for-profit online magazine, and ‘Waatea News’, a bilingual radio station, that provide written summaries of their radio broadcasts on their website were selected based on their prioritization of delivering stories that reflect Māori realities in AoNZ. The databases of both sources were searched from their inception in 2014 up to July 2023. Initial searches yielded 2164 texts (89% from Waatea News). Sleep was rarely the primary topic, mentioned implicitly or in passing reference in the context of other topics, including history, art, and politics. The articles were screened and exclusion criteria applied. These included duplicate articles and articles where sleep-related ‘te reo Māori’ (Māori language) words produced alternate meanings—e.g. ‘moe’, the te reo Māori word for ‘sleep’ yielding results including English name ‘Moe’ or the names of ‘iwi’ (tribe) and ‘rohe’ (tribal geographic area) containing ‘moe’. A decision tree containing keywords and initial and final yields are summarized in Fig. 1 (By D. Haami, 2025).

#### E-Tangata database search

During the initial search, the keyword ‘sleep’ yielded 77 articles, remaining unchanged with the application of the exclusion criteria. Results were also unchanged when using English variants of ‘sleep’, such as ‘asleep’ or ‘sleeping’. Follow-up searches were conducted using related English and te reo Māori terms: ‘moe’ (sleep) (initial *n* = 33, final *n* = 19), ‘bed’ (initial *n* = 96, final *n* = 4), and ‘slept’ (initial *n* = 28, final *n* = 12). In total, 112 articles were identified for further analysis (see Fig. 1).

#### Waatea News database search

The initial search using the keyword ‘sleep’ yielded 180 articles. Using the exclusion criteria, this was reduced to 130 articles. Follow-up searches were conducted using related English and te reo Māori variants: ‘moe’ (initial *n* = 532, final *n* = 2), ‘bed’ (initial *n* = 1188, final *n* = 1), ‘slept’ (initial *n* = 8, final *n* = 7), ‘asleep’ (initial *n* = 12, final *n* = 10), and ‘momenta’ (initial *n* = 22, final *n* = 8). A total of 158 articles were identified for analysis (see Fig. 1).

### Data analysis

The 270 online media articles retained from E-Tangata (41.5%) and Waatea News (58.5%) were compiled for analysis. Reflexive thematic analysis is a well-defined qualitative methodology that has been utilized across a diverse range of disciplines to identify meaning and patterns across data (Braun and Clarke 2006, 2019, Braun *et al.* 2022). A major strength of reflexive thematic analysis is its flexibility, allowing it to be utilized with diverse methodologies, including Indigenous methodologies, such as Kaupapa Māori. According to Braun and Clarke (2006), reflexive thematic analysis expects the researcher to engage critically and transparently in all parts of the research, clearly communicating all ‘assumptions about the nature of the data, what they represent in terms of “the world”, [and] “reality”’ (p. 81). While those operating from within a WEIRD (Henrich *et al.* 2010) and WASP (Berry 2015) worldview may see the transparent presentation of the subjective as a weakness of ‘scientific’ research, from an Indigenous perspective, the subjective is an advantage, especially when the researcher shares the same cultural background as the participants, with this insider’s perspective adding depth, nuance, and richness to the analysis and interpretation of the data (Bishop 2021).

Copies of the articles were added to NVivo (a qualitative analysis software system). Initial automated coding based on the keywords was used to expedite identifying sections of text relevant to sleep. Semantic content of the data drove the construction of more specific codes to represent meanings. For example, by automatically picking up words such as ‘sleep’ and ‘bed’, codes such as ‘sleeping rough’ or ‘safe infant sleep’ were identified. These were analysed further with a focus on semantic themes, patterning across texts, and social interpretations, resulting in the identification of three overarching topics: sleep as routine for health, production, and whānau wellbeing (e.g. sleep presented as a basic human right, linked to productivity, and research-based recommendations for improving sleep); sleep as a time of vulnerability (e.g. overcrowding, infant sleep safety, and the physical and psychological vulnerability of sleep); and sleep as a time for communal and cultural connection (e.g. cultural, communal, and intergenerational sleep practices). These were further refined using Durie’s (1985) Te Whare Tapa Whā (the four-sided house) model of wellbeing (George *et al.* 2021). Te Whare Tapa Whā describes four ‘taha’ (sides of the house, or dimensions) of health: ‘Taha Wairua’ (spiritual dimension), ‘Taha Hinengaro’ (psychological dimension), ‘Taha Tinana’ (physical dimension), and ‘Taha Whānau’ (family dimension). The foundation on which the ‘whare’ (house) stands is the ‘Taha Whenua’ (the physical environment), reflecting the importance of the wider social and natural environment on wellbeing. Using Te Whare Tapa Whā ensured the privileging of a Te Ao Māori lens in the construction and presentation of the themes.

## Results

**Figure 2:**
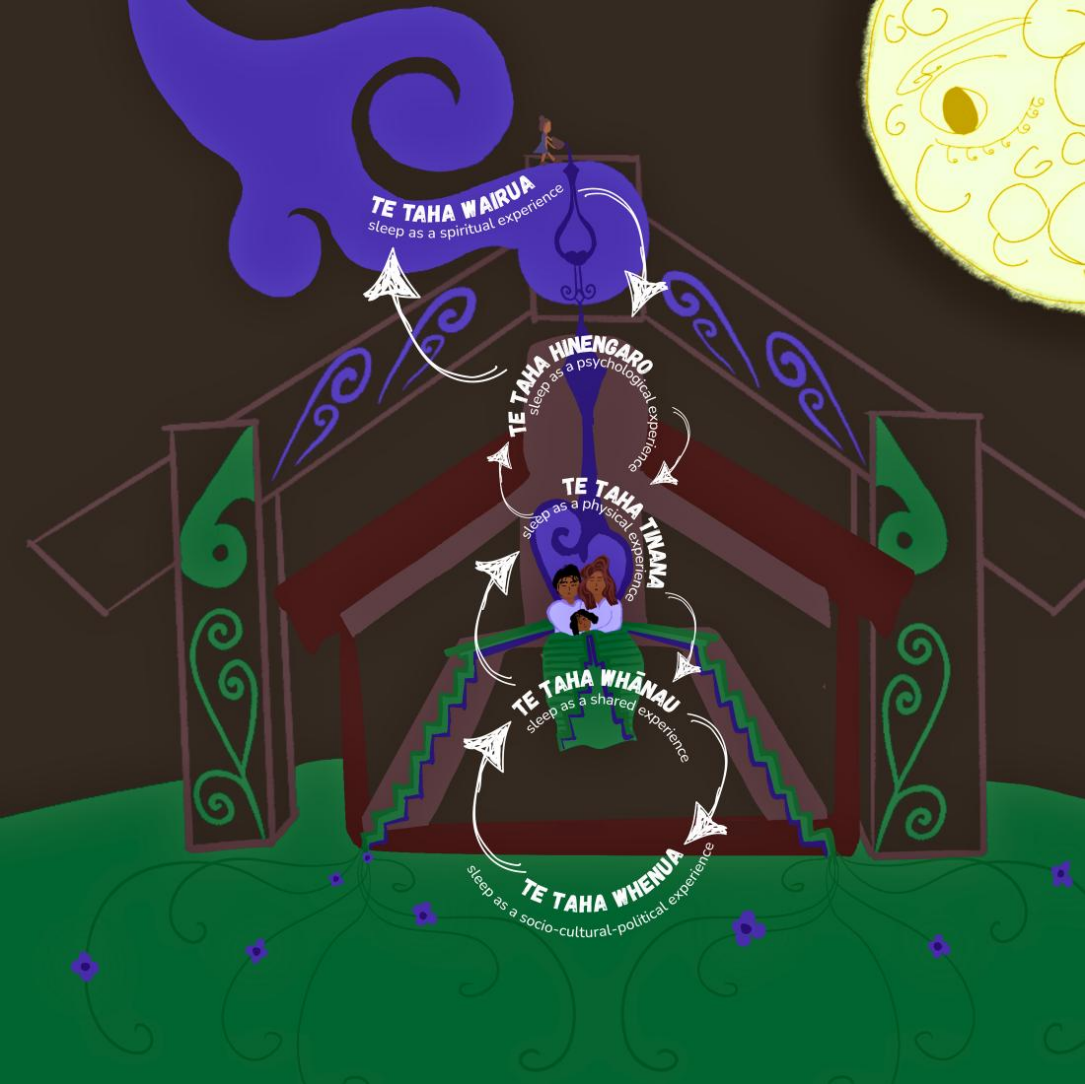
Applying Te Whare Tapa Whā to social constructions of sleep in Māori media.

Overall, five themes were identified: (i) Te Taha Wairua, sleep as a spiritual experience; (ii) Te Taha Hinengaro, relationship between sleep and psychological wellbeing; (iii) Te Taha Tinana, relationship between sleep and physical wellbeing; (iv) Te Taha Whānau, sleep as a shared experience; and (v) Te Taha Whenua, a place and space to sleep. Although the five themes have been structured separately in accordance with their associated taha, it is important to remember that each taha, and its state of wellbeing, impacts the wellbeing of the other taha, which, in turn, impacts sleep and sleep health. This interconnectedness between the taha was reflected across the data and has been purposefully incorporated into the writing of this section. Figure 2 (By D. Haami, 2025) provides a visual representation of a Te Whare Tapa Whā framing of sleep, which was developed during the analytical process.

### Te Taha Wairua—sleep as a spiritual experience

The relationship between the Taha Wairua and sleep was often described as an opportunity for traversing into ‘Te Ao Wairua’ (the spiritual dimension of reality). These was demonstrated in the variations of the phrase ‘moe mai rā’, which can be translated in numerous ways, including eternal sleep, rest in peace, to rest in the hands of the ‘atua’ (divine beings from beyond), and the final sleep.

*Moe mai ra e te rangatira, moe mai to moengaroa, moe mai i te ringaringa o te Atua (Sleep peacefully, O Chief, sleep peacefully in your everlasting rest, sleep peacefully in the hands of God)*.

These diverse conceptualizations can be directly linked to the multitude of subtle and intersecting meanings of the word moe, which includes being asleep, dreaming, literally having the eyes closed, cohabitation (or marriage), and reproduction. In this sense, moe, as a state of sleeping and dreaming, acts as a pathway for engaging in ‘matekitetanga’ (refers to engaging with the unseen, sickness, or the dead). Such engagements had a positive impact on the waking lives of individuals, providing a cathartic and healing experience that supported the wellbeing of their Taha Hinengaro and Taha Wairua.

*I am the notorious dreamer. I’ve imagined new worlds, old ones, people I’ve never met. I speak to ancestors in my sleep and have been comforted in burying my tears in their images*.

Sleeping and dreaming was also seen as an opportunity for creativity through matekitetanga. For example, the creation of ‘taonga’ (referring to something precious, a treasure) containing vital mātauranga can support the connection of future generations to Te Ao Māori, as exemplified in oral traditions such as ‘waiata’ (songs).

*Or it could be two o’clock in the morning and you wake up and you’ve got this tune, or you’ve got some words. I know other people who say they wake up and they’ve got a tune but they don’t do anything with it. They just go back to sleep and then forget it. Or they’re dreaming and there’s a tune there. But what you can do is you can record yourself. Quickly record it and then go back to sleep. And then wake up and put some words to it*.

Such engagement also ensures the continued transmission of mātauranga, te reo Māori, and tikanga to the next generation, maintaining the wellbeing of the Taha Whānau.

*One of the best things I ever did to pass on te reo Māori, was to sing to my children before they went to sleep—in particular a waiata whakaoriori* [a type of oral tradition containing mātauranga and whakapapa] *I wrote for them that speaks of their maunga* [mountain] *and moana* [body of water]*, their heritage as peacemakers, and their links to the past and present*.

### Te Taha Hinengaro—relationship between sleep and psychological wellbeing

The way in which the relationship between sleep and the Taha Hinengaro was predominantly informed by WASP research focused on the relationship between sleep duration and psychological wellbeing.

*They found a third of pregnant Māori women are sleeping less than six hours in their last trimester. They linked that to rates of pre-natal depression at least as high as that of post-natal depression. Just over one in four Māori women and one in seven non-Māori women screened during pregnancy had signs of clinical depression*.

As well as reported statistics such as the example above, tere were also descriptions of the lived experiences of the Taha Hinengaro and sleep.

*Not long after we noticed these first signs, our boy had what we now know was a depressive episode. He didn’t want to get up, and would sleep the entire day and most of the night, only waking from 10pm to 3am. He didn’t want to talk about anything or to anyone, and he kept to himself.*

However, there were several articles that extended this understanding beyond WASP notions of sleep, identifying the importance of understanding the interconnectedness between all dimensions of wellbeing.

*He hadn’t slept in several days, and he was in an incredible state of distress. He left his address without approval to see his kaumātua* [respected elder] *for spiritual guidance because his mother had started appearing to him*.

In the above excerpt, the compromised wellbeing of the Taha Hinengaro for this individual diminished their ability to safely navigate between Te Ao Wairua and ‘Te Ao Kikokiko’ (the physical realm). However, with the right support and guidance from the Taha Whānau, his Taha Hinengaro was strengthened.

### Te Taha Tinana—relationship between sleep and physical wellbeing

The relationship between the Taha Tinana and sleep was predominantly informed by WEIRD research focusing on the impact of sleep duration on physical wellbeing.

*Get eight hours of sleep every night. Both your brain and your body need it*.

Narrative threads demonstrating the interconnectedness between the different dimensions of wellbeing were also present, such as the impact of inadequate sleep on the Taha Whānau.

*Almost a third of Māori aren’t getting enough sleep. Researchers from Massey’s Sleep/Wake Research Centre have found 29 per cent of Māori and 22 per cent of non-Māori sleep less than seven hours a night on days when they work or study. A third of the short sleepers try to catch up with sleep on the weekends, causing what is known as social jetlag*.

Poor sleep associated with a compromised Taha Tinana—exhibited through serious medical conditions, such as sleep apnoea—was also identified as impacting both the Taha Hinengaro and Taha Whānau.

*The only thing that buggers up my theory are people with sleep apnoea. It’s the kind of snore that rises to a pitch and then suddenly stops as the breath gets taken away. The silence hangs in the air, and you’re not sure if they’re ever going to breathe again. Several seconds later, the snoring resumes, only to stop once more. Each subsequent gap seems longer, and each silence more nerve-wracking*.

### Te Taha Whānau—sleep as a shared experience

The relationship between the Taha Whānau and sleep was often described as an intergenerational experience. The predominant focus of many of these articles was SUDI, demonstrating the depth of the connection between Taha Whānau and Taha Whenua.

*The number of SUDI deaths has fallen from over 250 to about 50 to 70 a year through getting the message across that babies need to sleep on their backs and in some cases in safe sleep devices, but most of those who die are Māori or Pasifika babies*.

Messaging related to Kaupapa Māori solutions—specifically the creation of the ‘wahakura’, or pēpi pod, which protects the infant and allows for safe communal sleeping—accompanied these articles. This solution positively impacted not only the Taha Tinana of the infants but also the Taha Hinengaro and Taha Wairua of the wider whānau.

*They’re our kuia* [grandmothers]*, they’re our aunties, they’re our uncles who are leading the way and we’re centralising that mātauranga with them so while they teach whānau to weave they are also sharing that safe sleep message, to place baby on its back…so we know all those key messages are being interwoven while actually weaving the wahakura with whānau as well*.

The role of the wider whānau was also present in the intergenerational transmission of beliefs and routines regarding sleep.

*My nanny always said: “If you want sleep, you wait until you’re dead. Plenty of sleep when you’re dead.” So that’s what we were brought up with. And with Dad being all over the place, well, we got used to that. We’d go with him all over the country. At times, Dad never slept and neither did we*.

These intergenerational beliefs, which frame less sleep as positive, not only contradicts WASP and WEIRD research regarding what is considered as ‘good’ sleep but also reflects how the Taha Hinengaro can shape and influence the Taha Whānau.

### Te Taha Whenua—a place and space to sleep

The physical environment in which sleep occurred was a focus for many of the articles. The night-time environment was predominantly described as a time of vulnerability for the Taha Tinana and Taha Hinengaro. This vulnerability was notably amplified when the environment was physically, psychologically, and socially unsafe, as exemplified in situations of sleeping rough.

*She says there is little compassion or tolerance evident in the community, with homeless people being verbally and physically abused on the street, and rough sleepers are being trespassed from public areas without being given an alternative*.

There was also acknowledgement that this is a challenge not just in terms of physical locations, but the sense of stability and community that having a safe space to sleep provides, showing a strong link between the Taha Whenua and Taha Whānau.

*It’s about the children allowing them to stay where they are and not be transient. To be able to have a sense of community. All those things that build our kids up and give them a sense of pride of where they come from, but you can’t do that if you don't have a stable home, if you don't know where you're going to sleep that night or if you're coming to school or to daycare in a car that you slept in the night before.*

However, this vulnerability was not exclusively concerning those without homes. For whānau who do have a physical space to sleep indoors, the economic and political climate, i.e. the wider Taha Whenua, can intrude upon their home—their Taha Whenua—compromising the wellbeing of their Taha Whānau.

*We live in bigger families, we live closer together, we sleep closer together. Ultimately overcrowding is one part of the picture in terms of spread of group A strep and therefore the risk of rheumatic fever, a kid with a cough sleeping with a whole bunch of other kids, that kid will spread group A strep. If that kid has group A strep, then you’re spreading it amongst a whole bunch of kids*.

Other articles counteracted these narratives, focusing on how a safe and stable Taha Whenua can uplift the other dimensions of wellbeing. This was exemplified in stories exploring communal sleeping arrangements in culturally significant sites, such as ‘marae’, a space of communal living for Māori, where the ‘ārai’ (the veil) separating Te Ao Wairua and Te Ao Kikokiko is thin. The construction of the marae to enable this navigation of two realms originates in the mātauranga embedded in pūrākau (a narrative form of oral tradition used to codify and transmit knowledge across generations) (Lee 2009), such as ‘Noho Tatapū’ (which refers to residing in a state of restriction). Noho Tatapū narrates the origins of the universe through the story of ‘Ranginui’ (the Sky Father and representation of Te Ao Wairua), ‘Papatūānuku’ (the Earth Mother and representation of Te Ao Kikokiko), and their many children, who lived for aeons within the dark space existing between the tight embrace of their parents—between Te Ao Wairua and Te Ao Kikokiko.

*Visiting the exhibition is like being invited to a wānanga on Māori worldview. At the entrance stands a sentinel work by Fred Graham, one of the first generation of contemporary Māori artists who emerged in the 1950s. In his carving, the children of Rangi and Papa sleep in their parents’ embrace*.

Collective sleeping on the marae, close to the ārai, was essentially represented as fortifying the wellbeing of each taha, ensuring individuals and whānau not only safely navigate their sleeping and waking realities once they leave the marae but also are filled with energy and vitality for navigating their future pathways.

*I was 15, and although we’d visited our whānau in Aotearoa throughout my childhood, I’d never heard any conversations in te reo. I’d never been to a marae, let alone one of my own marae. And I had never seen and touched a tūpāpaku* [deceased person] *before, let alone slept beside one. It was a surreal experience. And it sparked a flame, a curiosity inside of me, to know more about my identity and my history, and to find that sense of belonging, of community and whānaungatanga* [extended family relationships].

The safety the marae provides is also vital for returning the compromised Taha Whenua of individuals and whānau to wellbeing, ensuring that night-time and sleep are no longer times of vulnerability for them, and enabling the restoration of the Taha Wairua.

*The kids will be our main focus we’re not taking rough sleepers. It’s a lot more intimate but we’re going to be focussed on quality outcomes rather than quantity. When our families come through we will do our best to whakakaha ake te mana te tapu te wairua o te whānau* [strengthen the life essence, the sacred spirit of the family].

The role that place and space play in sleep and wellbeing of individuals and whānau was consistently described across the articles, highlighting the importance of a stable Taha Whenua for attaining good wellbeing across ‘all’ dimensions of Te Whare Tapa Whā and good sleep—pai te moe, pai te ora.

## Discussion

Sleep as a sociocultural practice in AoNZ requires a greater understanding of Mātauranga Māori. Before colonization, such understandings were widely known and available through oral traditions, such as pūrākau. And although this Mātauranga Moe is not as widely known as it once was, the present research provides the foundation for its reclamation by demonstrating how, despite colonization, the essence of the Mātauranga Māori understanding of sleep continues to be transmitted intergenerationally. However, there is a layer of complexity to this, as the articles themselves, while created with a by Māori for Māori focus, are still influenced and shaped by the WEIRD and WASP paradigm. The mediascape itself is derived and based on Western journalistic expectations, while the journalists and writers developed their skills and understandings of ‘good’ media communication within Western educational institutions. Finally, the majority of the knowledge used as the basis for the media articles was derived from WEIRD and WASP research, which prioritizes Western, scientific, settler-colonial understandings of sleep. All these layers demonstrate the complexities and nuances of being and living Indigeneity in a colonized contemporary reality and the challenges faced in the decolonization, re- indigenization, and reclamation process.

Across the articles, the Taha Wairua was identified as the most important dimension of wellbeing, prioritized above any of the physical or psychological health benefits of normative sleep routines touted in WEIRD and WASP research. This reflects how wellbeing is understood within Mātauranga Māori, whereby both wellness and illness originate within the Taha Wairua—thus, if the Taha Wairua is well, all other dimensions of health will also be well (Haami *et al.* 2024b). While some of the media articles represented sleep as a routine and expected part of daily life, many referred to culturally specific practices and spaces where Māori may experience sleep differently to what is positioned as the ‘norm’ within a WEIRD worldview. For example, when engaging in ‘wānanga’ (a process of intergenerational collective thinking, discussion, problem solving, and knowledge contribution) (Smith *et al.* 2019), ‘tangihanga’ (traditional Māori funeral rites), or other gatherings where people sleep in a communal space, such as a marae ‘wharenui’ (central meeting and sleeping space) or ‘wharepuni’ (sleeping house), it was normal to have less sleep, more sleep interruptions, or a different sleeping and waking routine. Rather than being a problem, these differences were described in positive terms, affirming elements of cultural identity, reinforcing traditions and cultural practices, and uplifting the Taha Wairua. In addition, a sleep disrupted by vivid dreams (often involving deceased whānau members) was not necessarily presented as a ‘bad’ sleep, especially if it sparked creativity or improved the wellbeing of the Taha Wairua, Taha Hinengaro, or Taha Whānau. Thus, the boundaries between sleep, dreaming, and spiritual experiences, as represented in online Māori media content, was more blurred and accepted compared to Westernized discourses, reflecting the continued influence of Mātauranga Māori understandings of sleep as an inherently wairua state of being (Lindsay *et al.* 2022).

The bidirectional relationship between sleep and the Taha Hinengaro well established in WASP research was also identified (Lee and Sibley 2019). However, it was interesting to note that Māori were represented in many of the media articles as being more vulnerable to this relationship, including being more likely to be exposed to psychological conditions that result in less or lower quality sleep. The bidirectional relationship between sleep and the Taha Tinana, also well established in WEIRD research (Stewart *et al.* 2020), was also represented. Again, Māori were positioned as being more vulnerable and more likely to be exposed to physical conditions or health problems that result in less or lower quality sleep. The positioning of Māori in this way reflects the extent to which Western, colonial paradigms continue to underpin media communication systems, structures, and practices, shaping and perpetuating the negative framing Māori often face in the media (Moewaka Barnes *et al.* 2012). While previous research has identified Māori as being overrepresented in the space of psychological and physical ill health (Browne *et al.* 2006, Wilson *et al.* 2019), the genesis of this to colonization is generally not conveyed, and this was true for the articles analysed in this study. This exclusion from media communications reflects the extent to which colonization continues to influence these spaces of knowledge dissemination, whereby expectations of ‘good’ journalism are controlled by WEIRD (MacDonald and Ormond 2021), by white, settler-colonial perspectives and assumptions. It also demonstrates the importance of being critical consumers of media, able to examine all parts of the construction of media to understand the specific lens through which it has been developed and the specific narrative that is being privileged (Nairn *et al.* 2012).

Many of the examples from online Māori media sources were Taha Whānau focused. The benefits of communal sleeping arrangements were described within the context of the socioeconomic realities of overcrowded housing, creating a juxtaposition. On the one hand, the fact that Māori are more likely to have larger families and to live in smaller, overcrowded houses highlights the continued presence of systemic colonization —the origin of many of the social issues, such as poverty, that Māori continue to be overrepresented within. On the other hand, the sense of belonging, connectedness, and whanaungatanga experienced in such conditions is experienced as positive. Additional cultural variations around Māori preference for communal sleep and familial sleeping arrangements, such as bed-sharing, were also noted, whereby the joy and comfort of sharing beds or bedrooms with family members, the ease of attending to a baby when they sleep beside you, and the unique cultural benefits of sleeping overnight at the marae were presented as beneficial to health.

The location or environment in which we sleep—the Taha Whenua—underpins all sleep experiences (Boulton *et al.* 2022). The texts reflected the statistics concerning how many Māori do not have a safe place to sleep. Here the texts illustrated cases of Māori as more likely to find themselves ‘sleeping rough’, whether that be on the streets, in vehicles, or in unsafe, cold, damp, and overcrowded living conditions. The texts also focused heavily on SUDI and the unsafe conditions in which pēpi sleep. The collated texts on these subjects at first made for grim reading. However, many took on a more hopeful and positive tone through the presentation of Māori-driven solutions, with the principle of ‘manaaki ki te tangata’ (to care for, and be hospitable of, all people) apparent in the texts.

### Limitations and future directions

By including the reo Māori word for sleep—‘moe’—we captured some articles that were not specifically sleep related. While ‘moe’ is often directly translated to ‘sleep’, it is also used to refer to death, dreaming, cohabitation (or marriage), and reproduction. The multidimensional and layered way in which sleep is conceptualized and ‘languaged’ within Te Ao Māori demonstrates the importance of having an in-depth understanding of Mātauranga Māori to fully appreciate the nuances of sleep as an experience and state of being. Though this navigation of the complexities of te reo Māori did influence our analysis, when viewed through a critical Kaupapa Māori lens, it can be seen as a strength rather than a limitation. Within WEIRD and WASP, the English language is positioned as the expectation for sense-making, thus influencing expectations of what a limitation ‘should’ be and the rigidity of simplistic definitions of complex concepts. Exploring the wider social impacts of the WEIRD and WASP informed way of messaging and framing sleep through research examining audience responses to such language use across media is vital.

Our focus was limited to written media and sources considered to be ‘news’ pieces. While these may have been the most readily accessible type of media, it did mean that other types of discourse, including oral traditions and visual representations, were excluded. While oral traditions, such as pūrākau and ‘mōteatea’ (lament, traditional chant), continue to be challenging to access, visual representations of the sleep experience are more readily available and accessible online through search engines and digital archives. There are also numerous visual artefacts created by Māori visual artists inspired by the knowledge embedded within oral traditions. While reviewing the literature, it was identified that an examination of visual representations of sleep has yet to be undertaken. Thus, exploring this avenue in future research would not only fill this important gap but also contribute to the reclamation of Mātauranga Moe and challenge the dominance of WEIRD- and WASP-informed sleep discourses that predominate media in AoNZ.

## Conclusion

Sleep for Māori is a holistic experience—vital to the Wairua, Hinengaro, Tinana, Whānau, and Whenua dimensions of health. Though the oral traditions that were once present across all dimensions of Māori lived reality may not be as prevalent or accessible as they once were, the foundational Mātauranga Māori understanding of sleep continues to be transmitted intergenerationally. This research has essentially provided the foundation for the continued reclamation and re-privileging of this knowledge, as well as highlighting the importance of ensuring continued critical reflexivity and decolonial education for those who work within health promotion and are responsible for the creation and dissemination of health messaging. Through using Te Whare Tapa Whā, a model of wellbeing derived from Mātauranga Māori, this research provides a more appropriate and useful way of understanding sleep, which aligns with Māori worldviews specifically and Indigenous peoples more broadly, ensuring a more representative foundation on which future research can be undertaken and the Mātauranga Moe that was concealed through the colonizing process can be brought into the light once more.

## Supplementary Material

daag034_Supplementary_Data

## Data Availability

The data that supports the findings of this study were derived from publicly available media sources Waatea News and E-Tangata.

## Supplementary material

Supplementary material providing English translations for Māori language words and terms is available at *Health Promotion International* online.
