# Supplementary material for: Pai te moe, pai te ora: exploring the sociocultural practice of sleep in Aotearoa New Zealand through Māori media
Source: Health Promot Int. 2026 Mar 17;41(2):daag034. doi: 10.1093/heapro/daag034 (PMC13017027; doi:10.1093/heapro/daag034)
Supplement: daag034_Supplementary_Data [file daag034_supplementary_data.docx]

**Glossary of te reo Māori Words and Phrases**

*Ako* – space and time for learning

*Aotearoa* – New Zealand

*Ārai* – the veil between Te Ao Kikokiko and Te Ao Wairua (see below)

*Atua* – divine beings from beyond

*E-Tangata* – the people

*Iwi* – tribal group

*Kaumātua* – respected elder

*Kaupapa Māori* – at its essence, privileges research that is by Māori, for Māori and with Māori

Kōrero – speeches

*Kuia* – female elders, grandmothers

Manaaki – the process of showing respect, generosity and care for others

*Manaaki ki te tangata* - to care for, and be hospitable of, all people

*Māori* - the Indigenous people of Aotearoa

*Marae* – a space of communal living for Māori

*Mātauranga Māori* - the extensive repository of knowledge of everything existing within the universe developed over thousands of years

*Mātauranga Moe* - repository of knowledge related to sleep

*Matekitetanga* – refers to engaging with the unseen, sickness or the dead

*Maunga* – mountain

*Moana* – body of water

*Moe* – sleep (but also: death/to die, dream, being asleep or literally having the eyes closed, sexual intercourse, cohabitation (or marriage) and reproduction)

*Moe mai rā* - eternal sleep, rest in peace, to go into the night, the eternal night, to rest in the hands of the atua

*Moenga* – bed

*Mōteatea* - lament, traditional chant, sung poetry

*Noho tatapū* - residing in a state of restriction

*Pai te moe, pai te ora* – with good sleep, comes good wellbeing

*Papatūānuku* – the Earth Mother and representation of Te Ao Kikokiko

Pēpi – baby

*Pūrākau* – a narrative form of oral tradition used to codify and transmit knowledge vital to the survival and wellbeing of Māori across generations

*Ranginui* – the Sky Father and representation of Te Ao Wairua

*Rohe* – tribal geographic area

*Taha* – sides or dimensions

*Taha Hinengaro* – psychological dimension

*Taha Tinana* – physical dimension

*Taha Wairua* – spiritual dimension

*Taha Whānau* – family dimension

*Taha Whenua* – the land or foundations beneath the house

*Tangihanga* – traditional Māori funerary rites

*Taonga* – referring to something precious, a treasure

*Tau* – attaining a state of stability, peace, and purpose

*Te Ao Kikokiko* – the physical realm

*Te Ao Māori* – Māori worldview

*Te Ao Wairua* – the spiritual dimension of reality

*Te Reo Māori* – the Māori language

*Te Whare Tapa Whā* – The Four Sided House

*Tikanga* - culturally informed protocols or practices developed to keep the collective safe across all dimensions of wellbeing

*Tohu* – guidance, signs

*Tūpāpaku* – deceased person

*Waatea/Wātea* - to be free, unencumbered, clear, open

*Wahakura* – flax bassinette

*Waiata* – songs

*Wairua* – the spirit and the spiritual dimension of lived reality

*Wānanga* - a process of the Taha Wairua and Taha Kikokiko, comprised of intergenerational collective thinking, discussion, problem solving and knowledge contribution

*Whakakaha ake te mana te tapu te wairua o te whānau* – strengthen the life essence, the sacred spirit of the family

*Whakaoriori* – chanted lullaby

*Whakapapa* – genealogical origins

*Whānaungatanga* – engaging in extended family relationships

*Whare* - house

*Wharenui* - central meeting and sleeping space

*Wharepuni* - sleeping house
